# Exercise interventions for sleep and cognitive dysfunction in shift workers: a systematic review of randomized trials

**DOI:** 10.3389/fpubh.2026.1762359

**Published:** 2026-02-06

**Authors:** Fahad Hamoud Algharbi, Shibili Nuhmani, Mohammed Alsubaiei, Alsayed Shanb, Maher Alquaimi

**Affiliations:** 1 Department of Physiotherapy, College of Applied Medical Sciences, Imam Abdulrahman bin Faisal University, Dammam, Saudi Arabia; 2 Medical Support Services, Royal Commission Health Services Program, Royal Commission for Jubail and Yanbu, Jubail, Saudi Arabia; 3 Department of Respiratory Therapy, College of Applied Medical Sciences, Imam Abdulrahman bin Faisal University, Dammam, Saudi Arabia

**Keywords:** circadian rhythms, cognitive function, exercise, night shift, shift work, sleep quality

## Abstract

**Background and objectives:**

Shift work disrupts circadian rhythms and contributes to poor sleep, cardiometabolic risk, and cognitive impairment, which is especially concerning in safety-critical professions. While exercise improves sleep and cognition in the general population, its effects in shift-working adults remain unclear. This review synthesizes randomized controlled trials (RCT) evidence on structured exercise training to determine its impact on sleep and cognitive outcomes in shift workers, and examines intervention characteristics, mechanistic pathways, and barriers to clinical translation.

**Materials and methods:**

Following PRISMA and Cochrane recommendations, six databases were searched from inception to January 2025 for RCTs involving adult shift workers and structured exercise (aerobic, resistance, combined, HIIT, or in-shift activity breaks). Primary outcomes were sleep quality, quantity, and continuity assessed using the Pittsburgh Sleep Quality Index (PSQI), actigraphy or polysomnography, and the Karolinska Sleepiness Scale (KSS), as well as cognitive performance and alertness measured by the Psychomotor Vigilance Task (PVT). Risk of bias (RoB 2.0) and methodological quality (PEDro) were assessed. Due to heterogeneity in interventions and outcome measures, results were narratively synthesized.

**Results:**

Ten RCTs (*n* = 420; 60% healthcare settings) predominantly evaluated aerobic or mixed modalities. Eight studies reported significant improvements in PSQI, total sleep time, sleep efficiency, or wake after sleep onset, although effect sizes and clinical relevance were inconsistent. Three RCTs demonstrated improvements in alertness, reaction time, and short-term memory, particularly when interventions were timed post-shift or delivered as supervised workplace sessions. Mechanistic evidence from six studies indicated circadian phase shifting, improved autonomic balance assessed by heart rate variability (HRV), and reduced inflammatory markers. However, 80% had “some concerns” or “high” risk of bias, and adherence barriers such as fatigue and irregular schedules were common.

**Conclusion:**

Structured exercise programs, tailored to shift pattern and individual chronotype, show promise for enhancing sleep and cognitive function in shift workers. Workplace-based, supervised interventions maximize adherence and real-world applicability. Future adequately powered RCTs with standardized outcomes, mechanistic markers, and sector-diverse samples are needed before guideline-level recommendations can be established.

**Systematic review registration:**

Identifier, PROSPERO CRD420250650538. https://www.crd.york.ac.uk/PROSPERO/view/CRD420250650538.

## 1 Introduction

Shift work, a prevalent feature of modern economies, requires working outside of traditional daytime hours. This schedule disrupts the alignment between endogenous circadian rhythms and external environmental stimuli, leading to circadian misalignment, a recognized public health concern (1, 2). Evidence consistently links shift work with adverse outcomes, including cardiovascular and metabolic disorders, impaired immune regulation, and diminished cognitive function (3–7). Sleep disruption is central among these risks: shift workers commonly experience fragmented sleep, insomnia, and excessive daytime sleepiness, with severity varying by shift rotation pattern and exposure duration (1, 8, 9). The consequences extend beyond health, contributing to impaired vigilance, slower reaction times, and increased occupational errors (10, 11). Given the prevalence of night- and rotating-shift schedules in healthcare, transportation, and manufacturing, the economic and societal burden of accidents, impaired workplace performance, and lost productivity is significant (1).

The mechanisms underlying these deficits are complex. Circadian misalignment alters endocrine and metabolic function and elevates inflammatory activity, with biomarkers such as interleukin-6 (IL-6) and C-reactive protein (CRP) consistently elevated in shift-working populations (2, 12). Autonomic imbalance further compounds risk: a reduction in heart rate variability (HRV) reflects persistent sympathetic activation, which correlates with poor sleep architecture and impaired cognitive performance (13, 14). In addition, rotating shift work has been shown to blunt the normal 24 h oscillation of cardiac autonomic control, resulting in altered sympathovagal balance across the sleep–wake cycle (45). Moreover, disrupted sleep architecture reduces time in slow-wave and rapid eye movement (REM) sleep stages critical for memory consolidation and psychomotor functioning (3, 15). Over time, neuroinflammation and oxidative stress associated with repeated circadian disturbance may accelerate neurocognitive decline (16).

Exercise training is among the most effective non-pharmacological strategies for mitigating circadian-related impairments. In the general population, structured aerobic and resistance activity improves sleep latency, increases restorative sleep stages, and enhances alertness and cognitive flexibility (17–19). Beyond sleep outcomes, regular exercise has been shown to reduce cardiovascular sympathetic tone and enhance parasympathetic modulation, contributing to improvements in heart rate variability and the restoration of normal 24 h oscillations of heart rate and blood pressure (17, 20, 21). The mechanisms are diverse: exercise-induced increases in core body temperature facilitate subsequent sleep onset via thermoregulatory cooling (22); endocrine effects such as growth hormone release and enhancement of brain-derived neurotrophic factor (BDNF) support synaptic plasticity and cognitive function (23, 24); and regular activity reduces pro-inflammatory cytokines such as TNF-*α* while enhancing immune recovery (25, 26). In addition, central nervous system fatigue following exercise has been proposed to strengthen sleep drive, while psychological effects, including reduced stress and anxiety, further contribute to improved sleep continuity and cognition (21).

Despite these promising mechanisms, clear evidence regarding exercise as an intervention for shift workers is lacking. Research in this population remains limited, with few randomized controlled trials (RCTs), heterogeneous exercise protocols, and mixed outcome measures. For example, large-scale analyses reveal that shift workers engage in up to 20% less moderate-to-vigorous physical activity than day workers, citing fatigue and irregular schedules as barriers to adherence (20, 27). However, this reduced leisure-time physical activity may reflect the occupational physical activity paradox: shift workers in physically demanding roles (healthcare, manufacturing) experience sustained elevated heart rate and limited recovery during working hours, which does not provide cardiovascular benefits and may impair autonomic recovery, unlike leisure-time exercise (28, 29). This occupational strain likely depletes capacity for discretionary exercise, highlighting why structured interventions must be designed as recovery-oriented rather than additional physical demands. Consequently, it is unclear whether the established benefits of exercise on sleep and cognition in general populations translate effectively into routine shift-working contexts.

This systematic review aims to synthesize current evidence from RCTs of structured exercise for shift workers, focusing on whether training improves sleep and cognitive outcomes, which intervention characteristics maximize benefits, and what barriers exist to implementation in clinical and workplace settings.

## 2 Materials and methods

### 2.1 Protocol registration and reporting

This systematic review was conducted according to the Preferred Reporting Items for Systematic Reviews and Meta-Analyses (PRISMA) 2020 statement and registered prospectively in the International Prospective Register of Systematic Reviews (PROSPERO: CRD420250650538). The research question used the PICOS strategy (P: adult shift workers (≥18 years) engaged in rotating, permanent night, or simulated shift schedules across any occupational sector; I: structured exercise; C: usual activity, wait-list, or non-exercise controls; O: sleep quality/quantity, circadian markers, and cognitive performance; S: RCT). The review addressed three core questions: (1) whether exercise improves sleep and cognitive outcomes, (2) which intervention characteristics maximize benefits, and (3) what mechanistic pathways and implementation barriers exist.

### 2.2 Eligibility criteria

Randomized controlled trials (RCTs) of structured exercise interventions among adult shift workers (≥18 years, rotating or permanent non-day shifts; sector-specific or simulated) were included. Interventions could be any exercise method, delivered in any format or setting, provided that exercise was a principal component. Comparators were usual activity, wait-list, or non-exercise controls. Primary outcomes were sleep quality (subjective/objective), circadian alignment, and cognitive function. Studies were excluded if they (1) focused on non-shift workers, (2) used non-randomized or quasi-experimental designs, (3) were non-peer-reviewed, or (4) were not published in English.

### 2.3 Search strategy

A comprehensive search was conducted across PubMed, Scopus, Web of Science, MEDLINE, EMBASE, and Dimensions from inception to January 2025, using the keywords- shift work, exercise, sleep, circadian rhythms, and cognitive outcomes. Search strategies were devised in consultation with a medical librarian. The search terms keywords included: “shift work,” “shift worker,” “night shift,” “rotating shift,” and “day shift.,” “exercise,” “physical activity,” “exercise therapy,” “physical fitness,” and “motor activity,” “inspiratory muscle training,” “respiratory muscle training,” “breathing exercises,” “pulmonary rehabilitation,” and “respiratory muscle strengthening,” “sleep quality,” “sleep duration,” “cognitive function,” “cognitive performance,” and “neurocognitive outcomes.” (Details of the search strategy in each database is available as Supplementary file S1).

### 2.4 Study selection and data extraction

Two reviewers (FA & SA) independently screened titles/abstracts assessed full texts for eligibility and resolved disagreements by consensus. The following data were extracted in a standardized data extraction form.

*Study characteristics*: Author(s), year of publication, country, study design, setting (e.g., laboratory, workplace), sample size, population characteristics (e.g., age, sex, occupation, type of shift work).

*Intervention details*: Type of exercise, frequency, duration, intensity, supervision, timing relative to shifts.

*Comparator details*: Type of control intervention.

*Outcome measures*: Specific tools or methods used to assess sleep quality, cognitive function, and secondary outcomes, along with their respective units and time points of assessment.

*Key findings*: Quantitative data for all relevant outcomes (e.g., means, standard deviations, effect sizes, confidence intervals) for both intervention and control groups, at baseline and post-intervention. For studies reporting multiple time points, the longest follow-up data was prioritized. For studies reporting multiple outcomes for the same construct (e.g., different measures of sleep quality), the most clinically relevant or commonly reported measure was selected.

### 2.5 Risk of bias assessment

Risk of bias was evaluated using the Cochrane Risk of bias 2.0 tool, which examined five domains: randomization process, deviations from intended interventions, missing outcome data, outcome measurement, and selection of reported results, with each study classified as having low risk, some concerns, or high risk of bias. Methodological quality was additionally assessed using the PEDro scale, an 11-item checklist evaluating internal validity and statistical reporting, to facilitate comparison with prior exercise and rehabilitation reviews. We acknowledge that Cochrane discourages reliance on quality scales and summary scores, therefore PEDro results were interpreted descriptively alongside RoB 2.0, and not used to determine eligibility or to weight findings, noting that summary scoring may mask domain specific sources of bias. Both assessments were completed independently by two reviewers (FA & SA), and disagreements were resolved by consensus.

### 2.6 Data synthesis and analysis

Meta-analysis was not conducted in our study due to statistical heterogeneity across studies in intervention type, outcome measures, and study populations. In addition, there were key differences in exercise timing (pre−/in−/post-shift), delivery context (e.g., laboratory vs. workplace), and underlying mechanisms assessed, such as circadian biomarkers. These differences made meaningful quantitative pooling inappropriate, since the resulting effect estimates would not represent comparable constructions. Instead, results were synthesized narratively, structured by outcome domain (sleep, circadian, cognitive), and further grouped according to (1) intervention modality, (2) timing relative to shift or circadian phase, and (3) participant or workplace characteristics, permitting a more valid interpretation of patterns and implementation relevance. Intervention characteristics were centrally reported and cross-compared, highlighting dose–response factors, session logistics, supervision level, and sector-specific barriers/facilitators necessary for clinical translation.

## 3 Results

### 3.1 Study selection and characteristics

**Figure 1:**
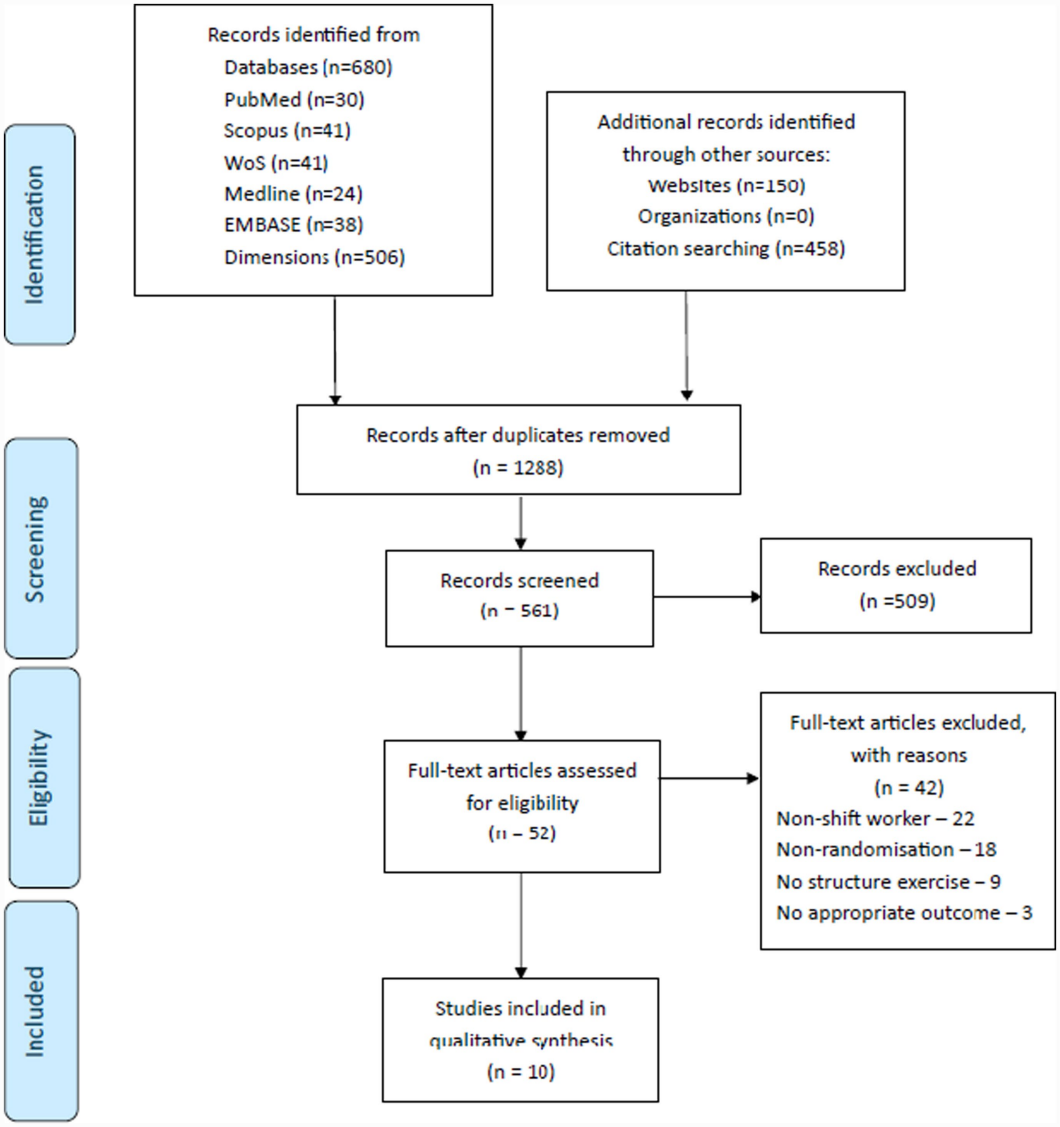
PRISMA flow diagram showing study selection process from initial database selections through final inclusion.

A total of 1,360 records were identified from database searches and other sources (see the PRISMA flow diagram in Figure 1). After removing duplicate articles, 561 records remained for screening. Of these, 52 full-text articles were assessed for eligibility, and 10 randomized controlled trials (RCTs) were included in the qualitative synthesis (30–39). All included studies were published from 1988 to 2024, with 60% published since 2020.

Table 1 provides an overview of included study characteristics. Across the 10 RCTs (*N* = 420 participants, mean sample size 42, range 18–75), 60% were conducted in healthcare settings and 70% of enrolled participants were female. Study designs consisted of eight parallel-group RCTs, one crossover RCT, and one cluster-RCT. Most studies enrolled real shift workers from hospital or industrial sectors, whereas two used simulated shift schedules. The majority of studies investigated rotating shift workers, with permanent night and simulated shifts each comprising 20% (Table 1 for detailed study and population characteristics).

**Table 1:** Characteristics of the included studies.

Study	Design	Population and shift exposure, schedule	Occupation	Intervention	Comparator	Sample size	Age (mean ± SD)/gender	Key findings
Atlantis et al., (30) (Australia)	RCT (parallel)	24/7-h worksite, 73% shift workers; rotating shifts, night shift dose: NR	Mixed shift workers	Aerobic + weight training; 3×/week; 24 weeks; supervised	Wait-list control	*N* = 32 (Ex = 14; Ctrl = 18)	31.0 ± 4.0 yrs.; Mixed gender	PSQI improvement: −2.1 ± 2.9 vs. −1.1 ± 2.6 control (*p* = 0.001). Women vs. Men: PSQI change: −4.6 points, *p* = 0.03. Non-shift workers: No significant change.
Barger et al., (31) (USA)	RCT (parallel)	Simulated shift work, healthy adults	Healthy volunteers, non shift workers	Daily aerobic cycling, 3 bouts/night, 45 min each, 7 consecutive nights	No exercise control	*N* = 18 (Ex = 9; Ctrl = 9)	23.0 ± 3.6 yrs.; Male only	Exercise delayed melatonin onset, offset, and midpoint (3.17 h) vs. controls (1.67 h) (*p* < 0.05) Timing-dependent effect, closer to natural melatonin onset the stronger effect (r = −0.73, *p* < 0.05)
Barger et al., (32) (USA)	RCT (crossover)	NASA flight controllers, night shift dose: 2 blocks of 5N consecutive	Aerospace	Light exposure + aerobic exercise, 10-min bouts at ≤65% HRmax, 3 bouts/shift, across 2 blocks of 5 nights.	Standard lighting + no exercise	*N* = 20	33.3 ± 8.0 yrs.; Mixed gender	KSS: Sleepier in control condition (*p* = 0.018) Reaction time improvement: 543.7 ms vs. 611.0 ms control (*p* = 0.031) VAS - experimental condition more alert (*p* < 0.0001)
Collins et al., (33) (Australia)	RCT (parallel)	Male rotational shift workers; 8–12 h rotating shifts, night shift dose: NR	Mixed sectors	Mixed-modality training, 3×/week, 12 weeks; semi-supervised	No exercise control	*N* = 27 (MICT = 9; RT = 10; Ctrl = 8)	MICT 41.0 ± 8.0 yrs.; Male only	MICT and RT: ↑TST post-night shift (MICT: *p* = 0.04, RT: *p* = 0.02) MICT: ↓CRP (*p* = 0.049)
Collins et al., (34) (Australia)	RCT (parallel)	Male rotational shift workers; 8–12 h rotating shifts, night shift dose: NR	Mixed sectors	Aerobic cycling (HIIT vs. MICT), single 30-min session	Active comparison	*N* = 26 (HIIT = 13; MICT = 13)	Mixed ages; Male only	MICT: ↓Sleep fragmentation (WASO) (*p* < 0.05) HIIT and MICT: ↑Anti-inflammatory response (↑IL-1Ra) (*p* < 0.016)
Easton et al., (35) (UK)	RCT (parallel)	Simulated night shifts, healthy non-shift workers, night shift dose: 5N consecutive	Simulated shift work	Light aerobic walking at 3.2 km/h, 3-min every 30 min, 5 consecutive nights	No exercise	*N* = 33 (Ex = 19; Ctrl = 14)	24.6 ± 4.8 yrs.; Female = 55%	Improved alertness in flexible (vs rigid) circadian type participants
Härmä et al., (36) Part I (Finland)	RCT (parallel)	Female shift workers (nurses), irregular shifts, night shift dose: 7D, 5E, 3N×/3-wk cycle	Hospital-based healthcare	Moderate aerobic training, 60%–70% HRmax, 2–6×/week, 4 months	No exercise	*N* = 75 (Ex = 49; Ctrl = 26)	Training 34.6 ± 6.8 yrs.; Female only	Increased sleep length post-evening shift (*p* < 0.05)
Härmä et al., (37) Part II (Finland)	RCT (parallel)	Female hospital shift workers (nurses), night shift dose: 7D, 5E, 3N×/3-wk cycle	Hospital-based healthcare	Moderate aerobic training, 60%–70% HRmax, 2–6×/week, 4 months	No exercise	*N* = 72 (Ex = 47; Ctrl = 25)	Female only	Decreased fatigue and increased alertness during night shifts
Niu et al., (38) (Taiwan)	RCT (parallel)	Female shift workers (nurses); poor sleep (CPSQI>5), night shift dose: D-shift during study	Hospital-based nurses	Aerobic exercise, 60 min/session, 3×/week, 8 weeks	No exercise	*N* = 60 (Ex = 30; Ctrl = 30)	26.0 ± 4.0 yrs.; Female only	Improved TST, SE at 4 and 8 weeks; lasting TST effects
Ha et al., (39) (South Korea)	Cluster RCT	Female shift workers (nurses); 8-h rotating shifts night shift dose: NR	Hospital-based nurses	Aerobic and resistance, 50%–60% Target HR, 2×/week, 12 weeks	Fitbit self-monitoring	*N* = 57 (Ex = 30; Ctrl = 27)	27.3 ± 3.0 yrs.; Female only	Improved sleep disturbance (*p* = 0.049), daytime dysfunction (*p* = 0.045)

Ctrl, control group; D, day shift; E, evening shift; Ex, exercise group; HIIT, high-intensity interval training; MICT, moderate-intensity continuous training; N, night shift; NA, not applicable; NASA, National Aeronautics and Space Administration; NR, not reported; PSQI, Pittsburgh Sleep Quality Index; RCT, randomized controlled trial; RT, resistance training; SD, standard deviation; SE, sleep efficiency; TST, total sleep time; WASO, wake after sleep onset; yrs = years.

### 3.2 Intervention characteristics

Interventions comprised aerobic-only exercise (6/10), combined aerobic-resistance (2/10), and multimodal (3/10) approaches. Sessions ranged from single bouts to programs of up to 24 weeks. Moderate-intensity aerobic exercise (60%–75% HRmax) was the predominant intervention modality, with only one trial (34) evaluating a high-intensity interval training protocol. Training frequency ranged from two to six sessions per week, with durations generally between 10 and 60 min. About half of the interventions were delivered in the workplace (supervised or semi-supervised), and the remainder were home-based or in laboratory settings. Exercise timing relative to shifts included pre-shift, during-shift, or post-shift delivery (Details of the interventions in Table 1).

When reported, compliance was assessed via training diaries, attendance records, heart rate monitoring during sessions, and wearable-derived tracking data such as accelerometer readings. However, several trials did not provide detailed adherence metrics.

### 3.3 Sleep outcomes

Sleep outcomes were assessed using subjective and objective measures (Table 2). Only one included trial employed polysomnography (PSG), the gold-standard method for objective assessment of sleep duration and architecture. In this study, Easton et al. (35) reported no significant changes in PSG-derived total sleep time or sleep architecture following the intervention, despite observing improvements in subjective alertness and reductions in sleepiness and fatigue.

**Table 2:** Effects of exercise interventions on sleep outcomes in shift workers.

Study	Outcome measure(s)	Assessment modality	Baseline	Post-intervention	Between-group difference	*p*-value	Direction of effect
Atlantis et al., (30)	PSQI	Self-reported	10.1	7.9	−2.1 (shift)	0.001	Improved (↓PSQI)
Härmä et al., (36) I	Sleep duration	Self-reported	6.2 h	7.0 h	+0.8 h	<0.05	Improved (↑TST)
Niu et al., (38)	TST, SE, WASO	Actigraphy	TST 383 min, SE 79%	TST 450 min, SE 84%	TST + 67 min, SE + 5%	<0.01, <0.05	Improved (↑TST, ↑SE)
Collins et al., (33)	TST, CRP	Actigraphy	TST 5.9 h, CRP 2.3	TST 6.5 h, CRP 2.0	TST + 0.6 h, CRP –0.3	0.04, 0.049	Improved (↑TST, ↓CRP)
Collins et al., (34)	WASO	Actigraphy	75 min	49 min	−26 min	<0.05	Improved (↓WASO)
Ha et al., (39)	PSQI	Self-reported	9.1	6.8	−2.3	0.025	Improved (↓PSQI)
Härmä et al., (37) II	Sleep quality	Self-reported	Fair	Good	Improved	<0.05	Improved (↑quality)
Barger et al., (31)	Melatonin phase		DLMO 01:30	DLMO 04:47	+3.17 h delay	<0.05	Delayed onset
Barger et al., (32)	Actigraphy, KSS	Actigraphy	6.7 h, KSS 5.3	6.9 h, KSS 4.7	NS	NS	No change sleep, ↑alert
Easton et al., (35)	PSG, sleepiness	PSG	6.5 h, KSS 6.7	6.6 h, KSS 5.2	NS	NS	No change sleep, ↑alert

CRP, C-reactive protein; DLMO, dim light melatonin onset; KSS, Karolinska Sleepiness Scale; NS, not significant; PSG, polysomnography; PSQI, Pittsburgh Sleep Quality Index; SE, sleep efficiency; TST, total sleep time; WASO, wake after sleep onset.

Eight of the 10 studies (80%) reported significant improvements in at least one sleep outcome following exercise intervention. Subjective sleep quality (PSQI) improved in three studies, with reductions ranging from −2.1 to −4.6 points compared to controls. Four studies reported increases in total sleep time (TST) (mean TST increases 20–70 min) based on actigraphy. Sleep efficiency improved in two studies, and wake after sleep onset (WASO) was reduced in three. Two studies, (32, 35), reported no change in sleep duration or architecture despite cognitive improvements.

Heterogeneity in outcome tools was substantial: PSQI and related subjective scales were used in 3 studies; actigraphy in 5 studies (with differing analytic criteria); and polysomnography in 2 studies (assessing different sleep architecture indicators). This variability precluded quantitative pooling and estimation of effect size. Minimal clinically important differences (MCID) for these outcome measures were not defined for shift worker populations. Additionally, PSQI findings should be interpreted cautiously as the instrument was not designed for shift-specific sleep patterns and may not reflect objective sleep continuity or circadian alignment (see Table 2 for individual study sleep outcome data and direction of effect).

### 3.4 Cognitive outcomes

Three studies (32, 35, 37) evaluated cognitive performance as a secondary outcome (Table 3). Reported domains included alertness (visual analog scale, Karolinska Sleepiness Scale), reaction time (psychomotor vigilance task), and short-term memory (SAM-test). Two studies (32, 37) found improved alertness and faster reaction times in exercise groups compared to controls. Barger et al., (32) reported significantly faster reaction times (mean slowest 10%: 543.7 ms vs. 611.0 ms, *p* = 0.031) with no difference in TST. Easton et al. (35) found reduced fatigue and sleepiness in flexible chronotypes exposed to activity breaks, though no objective sleep improvements were noted.

**Table 3:** Effects of exercise interventions on cognitive outcomes in shift workers.

Study	Domain(s)	Test/measure	Baseline	Post-intervention	Between-group difference	*p*-value	Direction of effect
Barger et al., (32)	Alertness, RT	VAS, PVT	VAS 4.9, RT 611.0 ms	VAS 5.8, RT 543.7 ms	RT − 68 ms (slowest 10%)	<0.031	Improved (↑) alert/RT
Härmä et al., (37) II	Alertness, STM	VAS, SAM-test	VAS 45, STM 18.2	VAS 67, STM 20.1	↑Alertness and STM	<0.05	Improved (↑)
Easton et al., (35)	Sleepiness, fatigue	KSS, fatigue scale	KSS 6.7, fatigue 22	KSS 5.2, fatigue 19	↓Sleepiness, ↓fatigue	NS	Improved (↑)

VAS, visual analog scale; PVT, psychomotor vigilance task; RT, reaction time; STM, short-term memory; SAM, search and memory; KSS, Karolinska Sleepiness Scale; NS, not significant.

All three studies used different cognitive batteries, limiting the comparability of results. Only alertness and reaction time domains showed consistent benefit; memory improvements were less consistently reported (see Table 3 for cognitive outcome details).

### 3.5 Mechanistic biomarkers (secondary outcomes)

Six studies reported mechanistic or biomarker endpoints. Circadian markers (melatonin phase) were assessed in two studies (31, 32), which observed exercise-associated delays in melatonin onset (2–3 h) using protocol-specific timing. Autonomic modulation was measured in two studies (38, 39) using heart rate variability (HRV), which found increased parasympathetic activity post-intervention. Markers of systemic inflammation (CRP, IL-6, TNF-*α*, IL-1Ra) were evaluated in five studies, with three reporting significant reductions or anti-inflammatory shifts post-exercise.

Heterogeneity in chosen biomarkers, sampling times, and laboratory methods prevented cross-study synthesis (Mechanistic outcomes are detailed in Table 4).

**Table 4:** Exercise-related mechanistic effects on circadian, autonomic, and inflammatory markers.

Study (year) and population	Exercise type, timing	Melatonin phase shift	HRV outcome	Inflammatory markers and others	Key findings/notes
Barger et al., (31) Simulated night shift (NASA)	Aerobic (cycling), during shift	Delayed onset/offset by ~3.17 h vs. 1.67 h in controls (timing-dependent)	NR	NR	Strongest shift when exercise timed near DLMO.
Collins et al., (33) Rotational shift workers	Mixed modality, post-shift	NR	NR	↓CRP (*p* = 0.049), non-significant ↓IL-6	Regular, moderate-intensity post-shift exercise.
Collins et al., (34) Rotational shift workers	HIIT vs. moderate intensity, post-shift	NR	NR	↑IL-1Ra (anti-inflammatory, *p* < 0.016), No change IL-6/CRP	Both HIIT and moderate exercise promoted anti-inflammatory effect
Niu et al., (38) Nurses, rotating shifts	Aerobic walking, post-shift	NR	↑ HRV (SDNN, *p* = 0.01)	↓TNF-α (*p* = 0.02), ↓CRP (trend)	Improved ANS balance and reduced inflammation at 8 weeks
Härmä et al., (36); Härmä et al., (37) Female nurses	Aerobic, supervised, mixed timing	NR	NR	NR	Protocol focused on sleep/cognitive, not mechanistic endpoints
Ha et al., (39) Nurses, rotating shifts	Aerobic + resistance, 12 weeks, post-shift	NR	↑ HRV (HF power, *p* = 0.04)	↓IL-6 (*p* = 0.04)	Significant increase in parasympathetic activity and reduced inflammation
Barger et al., (32) Night-shift NASA controllers	Aerobic, during shift	Delayed melatonin onset by ~2 h (timing effect), Phase shift	NR	NR	Phase shift correlates with exercise timing/chronotype
Atlantis et al., (30) Healthcare workers	Aerobic + resistance, 8 weeks, post-shift	NR	NR	↓CRP (trend), ↑IGF-1 (trend)	Slight anti-inflammatory and metabolic improvement
Easton et al., (35) Simulated night shift	Light aerobic breaks during night shift	NR	NR	NR	Mechanistic focus on alertness/cognition

ANS, autonomic nervous system; CRP, C-reactive protein; DLMO, dim light melatonin onset; HF power, High Frequency power; HIIT, high-intensity interval training; HRV, heart rate variability; IGF-1, insulin-like growth factor-1; IL-1Ra, interleukin-1 receptor antagonist; IL-6, interleukin-6; NR, not reported; SDNN, standard deviation of normal-to-normal intervals; TNF-α, tumor necrosis factor-alpha.

### 3.6 Risk of Bias and study quality

**Figure 2:**
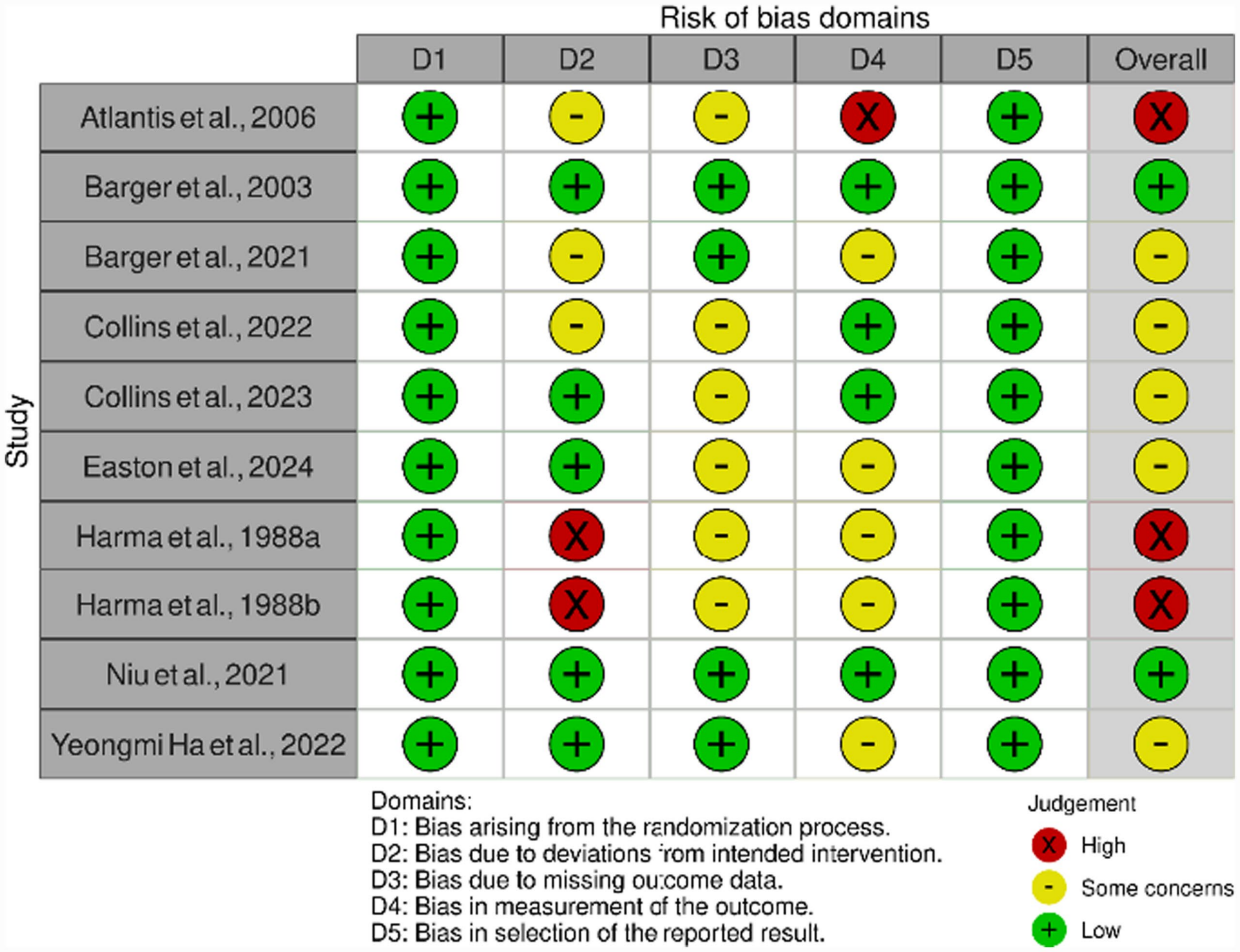
Risk of bias assessment (RoB 2.0).

Across the ten included studies, five (50%) were rated as some concerns (32–35, 39) and three were rated high for risk of bias (30, 36, 37), mainly due to lack of participant and therapist blinding and incomplete outcome data; two studies were rated as low risk (31, 38). The mean PEDro score was 6.7 (range 6–8), representing moderate methodological quality. Blinding of outcome assessors was inconsistently reported, and only three trials achieved high quality (PEDro ≥ 8/10). (Figure 2 and Table 5 for risk of bias and methodological quality details).

**Table 5:** PEDro scale assessment.

Study ID	PEDro Score (/10)	Criteria Met	Key notes/justifications for deductions
Atlantis et al., (30)	6	1–4, 9–11	Deductions for no blinding (S, T, A) and <85% follow-up; ITT done with last observation carried forward.
Barger et al., (31)	8	1–4, 7–11	Same pattern: no subject/therapist blinding, but excellent execution with concealed randomization, blinded lab assays, 100% completion.
Barger et al., (32)	8	1–4, 7–11	Lost points for no subject/therapist blinding; strengths: concealed randomization, baseline similarity, assessor blinding for objective tests, full retention, ITT.
Collins et al., (33)	6	1–4, 7, 10–11	No subject/therapist blinding; <85% data for actigraphy; no ITT; otherwise concealed randomization and objective blinded lab assessments.
Collins et al., (34)	6	1–4, 7, 10–11	Similar deductions as 2022: no blinding (S/T), incomplete actigraphy, no ITT; robust randomization/concealment and baseline comparability.
Easton et al., (35)	6	1–4, 8, 10–11	No subject/therapist/assessor blinding; no ITT; otherwise concealed randomization and high completion in main subgroups.
Härmä et al., (36) (Part I)	6	1–4, 10–11	Lost points for no blinding (S/T/A), <85% follow-up, and no ITT (per-protocol only).
Härmä et al., (37) (Part II)	6	1–4, 10–11	Same limitations as Part I substantial attrition, no blinding, no ITT analysis.
Niu et al., (38)	8	1–4, 7–11	Only missing subject and therapist blinding; strong methodology with concealment, blinded assessor, ITT, and high retention.
Ha et al., (39)	7	1–4, 7–8, 10–11	No subject/therapist blinding; no ITT analysis; otherwise strong design with concealed randomization and assessor blinding for objective PA data.

### 3.7 Data synthesis and heterogeneity

The Meta-analysis was not performed due to the high degree of heterogeneity in intervention protocols (modality, intensity, timing), delivery setting (lab, workplace, home), participant populations (rotating, permanent, simulated shift workers), and outcome measures (PSQI, actigraphy, PSG, VAS, PVT). This prevented effect size estimation and direct comparison between studies. All results are presented as a structured narrative synthesis.

## 4 Discussion

### 4.1 Summary of main findings

This systematic review of 10 randomized controlled trials (*n* = 420) examined structured exercise interventions for improving sleep and cognitive outcomes in shift workers. Despite 80% of studies reporting sleep improvements, the clinical significance remains uncertain given: (1) heterogeneous outcome measures preventing effect size estimation, (2) predominance of short-term interventions (<12 weeks) in 70% of studies, and (3) unclear minimal clinically important differences for actigraphy parameters in shift workers. Three studies demonstrated cognitive benefits, particularly alertness and reaction time improvements, though comprehensive assessment was limited. Post-shift exercise most consistently improved sleep consolidation, while during-shift exercise produced circadian phase delays. However, two studies (32, 35) found null sleep findings despite improvements in alertness, highlighting mechanistic complexity. Although polysomnography provides the gold-standard assessment of sleep, the only PSG-based study did not demonstrate changes in objective sleep duration or architecture (35).

The two trials using objective sleep assessment reported improved alertness despite no detectable changes in sleep duration or architecture. Easton et al. (35), the only PSG-based trial, found no significant changes in PSG-derived total sleep time or sleep architecture, yet participants reported improved alertness. Similarly, Barger et al. (32) showed improved alertness and reaction time without actigraphic sleep changes, though short wavelength light exposure co-intervened. This dissociation suggests exercise acts primarily as an acute alertness countermeasure rather than altering sleep physiology. Expectancy effects may also contribute. Exercise timing appears critical, post shift exercise may support sleep consolidation, whereas during shift exercise, particularly with environmental manipulations like light exposure, improves alertness and safety without measurable sleep changes. Future trials should separate exercise from light exposure and pair objective sleep with circadian phase markers.

This suggests that observed benefits may relate to alertness or fatigue-related mechanisms rather than measurable changes in sleep physiology; however, conclusions are limited by the small number of PSG-based studies. These findings are consistent with broader evidence showing that targeted sleep-promoting behaviors, such as circadian-informed lighting, melatonin supplementation, blue light blocking, and daytime napping, can enhance reaction time and cognitive efficiency in sleep-restricted conditions (40–43). The evidence base remains limited by small sample sizes (mean *n* = 42), short follow-up periods, and 80% of trials rated as having “some concerns” or “high” risk of bias, precluding meta-analysis.

### 4.2 Comparison with existing systematic reviews and unique contribution

Prior systematic reviews have established that exercise is effective for improving sleep in general populations (17), with reporting small-to-moderate effect sizes for sleep quality (Hedges’ *g* = 0.36 for regular exercise) across 66 studies. Similarly, Alnawwar et al. (44) documented that moderate-intensity physical activity reduces sleep latency and increases total sleep time in adults with sleep disorders. However, these reviews did not specifically address shift workers or examine exercise as a circadian realignment strategy, an important distinction given that shift workers experience unique physiological challenges, including circadian misalignment, chronic sleep restriction, and occupational safety demands absent in general populations.

Our review addresses this evidence gap by synthesizing RCT-only evidence specific to shift workers, revealing that 8 of 10 studies (80%) reported positive sleep findings comparable to the 70%–75% response rates observed in general population reviews (17). However, three key differences were identified that limit direct comparisons.

First, shift workers may respond differently to exercise timing: our findings suggest post-shift exercise serves a transitional role facilitating sleep onset, whereas during-shift exercise acts as an alerting countermeasure with phase-shifting properties (31, 32). Timing considerations of this nature are largely unaddressed in day-worker studies.

Second, two studies with null sleep findings (32, 35) nonetheless demonstrated significant cognitive improvements (alertness, reaction time), suggesting exercise may benefit shift workers through pathways independent of sleep restoration. Barger et al. (32) found significant alertness improvements (*p* < 0.0001) and faster reaction times (543.7 vs. 611.0 ms, *p* = 0.031) despite no change in actigraphic sleep duration (6.7 vs. 6.9 h, ns), indicating exercise may enhance daytime function via direct neurophysiological mechanisms rather than sleep-mediated recovery. Similarly, Easton et al. (35) observed that breaking up sitting with light-intensity walking improved early-night alertness in flexible chronotypes despite no polysomnographic sleep improvements, potentially explained by acute arousal effects and individual circadian tolerance differences.

Third, adherence barriers are heightened in shift workers: fatigue, irregular schedules, and competing recovery needs reduce physical activity levels by 20% compared to day workers (27), complicating intervention sustainability.

### 4.3 Critical analysis of heterogeneity and clinical translation

Meta-analysis was deemed inappropriate due to substantial heterogeneity across multiple dimensions: (1) intervention timing (pre/during/post-shift) targeting different mechanisms (alertness vs. circadian realignment vs. sleep consolidation), (2) delivery context (laboratory-based efficacy studies vs. workplace feasibility studies measuring different constructs), (3) outcome measurement (PSQI, actigraphy, PSG each capturing distinct sleep dimensions), and (4) participant characteristics (simulated vs. operational shift work; rotating vs. permanent nights). Pooling these heterogeneous studies would produce misleading effect estimates, diminishing the visibility of clinically meaningful subgroup differences.

For clinical translation, this heterogeneity indicates that exercise is not a uniform intervention timing, modality, and individual factors (chronotype, shift pattern) critically moderate effectiveness, necessitating personalized prescription rather than one-size-fits-all recommendations.

The inability to conduct meta-analysis due to measurement heterogeneity prevents effect size quantification, limiting our ability to define clinically meaningful benchmarks. For example, does a 30-min increase in actigraphic total sleep time translate to reduced occupational errors in safety-critical industries? Without standardized outcomes and minimal clinically important difference (MCID) thresholds specific to shift workers, the practical significance of observed sleep improvements remains unclear.

Across the ten included randomized controlled trials, risk of bias assessment indicated that eight studies (80%) were rated “some concerns” or “high” risk for overall risk, primarily due to challenges with participant and therapist blinding (*n* = 8) and incomplete outcome data (*n* = 5). Only two studies achieved low risk of bias across all domains. Methodological quality, evaluated using the PEDro scale, was moderate overall, with a mean score of 6.7 out of 10 (range 6–8 across studies). These limitations are common in behavioral intervention research, but they diminish confidence in effect estimates and raise the potential for performance bias, especially for subjective sleep and cognitive outcomes. While objective measures, such as actigraphy and laboratory-based cognitive tests, help mitigate some bias, the inability to fully blind most exercise protocols and variable adherence reporting remain important barriers to drawing reliable, generalizable conclusions. Greater methodological standards in future research especially complete follow-up, improved objective measurement, and transparent reporting is needed to strengthen confidence in study findings.

#### 4.3.1 Synthesis of findings by intervention characteristics

Synthesis across included trials revealed clear patterns regarding the most effective intervention characteristics for shift workers. Moderate-intensity aerobic training, consistently delivered 2–3 times per week in bouts of 30–60 min, produced the most marked sleep and cognitive improvements particularly when scheduled post-shift as a transition to sleep. Combined aerobic and resistance protocols offered similar benefits, though longer sessions and complexity posed adherence challenges. High-intensity interval training (HIIT) demonstrated promise for efficiency but requires further validation. Timing relative to shift was critical: during-shift exercise most effectively shifted circadian phase, while post-shift sessions optimized sleep outcomes. Workplace-based and supervised programs demonstrated the highest levels of adherence, highlighting the importance of organizational support and tailored implementation. Brief, repeated activity breaks, as reported by Easton et al. (35), were also effective in improving alertness and reducing fatigue during simulated night shifts, supporting practical application in demanding professional settings. These findings reinforce the need for customized, pragmatic exercise prescriptions to accommodate the diverse needs of shift-working populations.

This review’s strengths include comprehensive searching across multiple databases, inclusion of only RCTs to ensure causal inference, dual independent screening and quality assessment using Cochrane RoB 2.0 and PEDro scales, and strict adherence to PRISMA 2020 reporting guidelines. Importantly, this is the first systematic review that exclusively synthesizes exercise intervention evidence for shift workers, directly addressing a recognized research priority within occupational health.

However, substantial limitations constrain confidence in our conclusions:

- Small sample sizes (mean *n* = 42, range 18–75) limit statistical power and effect estimate precision, reflecting recruitment challenges inherent in workplace-delivered shift work trials with objective assessments. Consequently, many studies are underpowered to detect clinically meaningful changes, particularly for objective outcomes, increasing false negative risk.
- Gender imbalance and occupational bias (70% female predominance, with healthcare workers representing 60% of the evidence base) threaten generalizability to male-dominated shift sectors (transportation, manufacturing, emergency services) where work demands, fatigue profiles, and intervention feasibility may differ substantially.
- Unmeasured psychosocial workplace factors (job strain, workload, staffing levels, organizational support, work–life conflict) were not consistently measured or reported, creating potential confounding of sleep and cognitive outcomes and influencing adherence to exercise interventions.
- Inconsistent baseline health and exposure reporting, including obesity/BMI, hypertension, cardiometabolic comorbidities, medication use, and commuting time, limits ability to identify confounders and effect modifiers that may influence responsiveness to exercise.
- Co-intervention designs combining exercise with other interventions [e.g., short-wavelength light exposure in Barger et al. (32)] prevent isolation of exercise-specific effects on observed outcomes.
- Missing shift schedule specificity: Most trials did not report night shift frequency or rotation direction, limiting schedule-specific interpretation of sleep and alertness outcomes.
- Short follow-up periods (mode 8–12 weeks; none exceeding 24 weeks) prevent conclusions about intervention durability, a particularly critical gap given that shift work is often a long-term employment condition requiring sustained, long-lasting strategies.

### 4.4 Clinical and workplace implications

Clinicians managing shift workers with sleep complaints may suggest but should not prescribe moderate-intensity aerobic exercise (2–3 sessions/week, 30–60 min) as a complementary strategy, recognizing that evidence remains preliminary. Timing appears critical: post-shift exercise may facilitate sleep transition, while pre-shift exercise may enhance on-shift alertness, though individualization by chronotype and shift pattern is likely necessary. Importantly, the null sleep findings in Barger et al., (32) and Easton et al., (35) alongside positive cognitive outcomes suggest exercise may benefit shift workers even without measurable sleep restoration a finding with potential relevance for workers unable to achieve adequate sleep duration due to operational constraints.

Employers should view these findings as preliminary evidence supporting workplace exercise program feasibility but should not mandate participation given the limited evidence base (only 10 heterogeneous RCTs, *n* = 420 total, 80% with bias concerns). Workplace-based interventions (50% of studies) achieved higher adherence than home-based programs, suggesting organizational support (e.g., on-site facilities, protected time) may be important facilitators, though formal cost-effectiveness analyses are absent.

### 4.5 Research priorities

Research needs include:

1. Adequately powered RCTs with standardized, validated sleep outcomes (Pittsburgh Sleep Quality Index for subjective quality; actigraphy for sleep–wake patterns with agreed analysis protocols) and cognitive performance batteries (psychomotor vigilance task, sustained attention tests) to enable future meta-analysis and effect size quantification.
2. Sector-diverse sampling beyond healthcare to establish generalizability across transportation, manufacturing, emergency services, and public safety professions with differing work demands.
3. Long-term follow-up (≥6 months) assessing intervention sustainability, adherence trajectories, and whether benefits persist or require ongoing participation.
4. Mechanistic investigations incorporating circadian phase markers (dim-light melatonin onset), sleep architecture (polysomnography), autonomic function (heart rate variability), and inflammatory biomarkers to clarify why some individuals show sleep improvements while others show only cognitive benefits, and to identify who benefits most.
5. Personalization trials testing whether exercise prescription tailored to individual chronotype, shift pattern (rotating vs. permanent; forward vs. backward rotation), and baseline fitness produces superior outcomes compared to standardized protocols.
6. Implementation science studies evaluating workplace program uptake, barriers to participation (fatigue, time constraints, competing recovery strategies), cost-effectiveness, and critically whether exercise-induced cognitive improvements translate to reduced occupational errors and enhanced safety in operational settings.
7. Formal guideline development incorporating GRADE methodology, Delphi consensus processes, and stakeholder input (shift workers, employers, occupational health practitioners) is warranted once the evidence base matures to support specific recommendations. Until then, exercise should be positioned as a promising but preliminary strategy requiring further validation, not as evidence-based standard of care.

## 5 Conclusion

This systematic review provides preliminary evidence that structured exercise may improve sleep quality and cognitive function in shift workers, though substantial uncertainty remains. Moderate-intensity aerobic exercise (2–3 sessions/week) shows promise, particularly when timed post-shift for sleep benefits or pre−/during-shift for alertness enhancement. However, conclusions are constrained by small sample sizes (*n* = 420 across 10 trials), short follow-up periods, measurement heterogeneity preventing meta-analysis, and predominance of healthcare worker samples limiting generalizability. The evidence is insufficient to support specific prescriptive recommendations or formal clinical practice guidelines. Larger, longer-term, sector-diverse RCTs with standardized outcome measures, mechanistic assessments, and implementation science designs are essential to establish exercise as an evidence-based intervention for shift work-related sleep and cognitive impairments.

## Data Availability

The original contributions presented in the study are included in the article/Supplementary material, further inquiries can be directed to the corresponding author.

## Glossary

PSQI: Pittsburgh Sleep Quality Index
TST: Total sleep time
SE: Sleep efficiency
WASO: Wake after sleep onset
KSS: Karolinska Sleepiness Scale
DLMO: Dim light melatonin onset
PSG: Polysomnography
PVT: Psychomotor vigilance task
STM: Short term memory
SAM: Search and memory test
VAS: Visual analog scale
HRV: Heart rate variability
HF: High frequency (HRV component)
SDNN: Standard deviation of NN intervals
ANS: Autonomic nervous system
HIIT: High intensity interval training
MICT: Moderate intensity continuous training
RT: Resistance training
CRP: C reactive protein
IL 6: Interleukin 6
IL 1Ra: Interleukin 1 receptor antagonist
TNF *α*: Tumor necrosis factor alpha
IGF 1: Insulin like growth factor 1
