# Supplementary material for: Exercise interventions for sleep and cognitive dysfunction in shift workers: a systematic review of randomized trials
Source: Front Public Health. 2026 Feb 6;14:1762359. doi: 10.3389/fpubh.2026.1762359 (PMC12920463; doi:10.3389/fpubh.2026.1762359)
Supplement: Supplementary file 1 [file Table_1.DOCX]

**Appendix A**

Supplementary File 1: Search Strategy and Study Selection

Electronic Database Search Strategy

**Date of Search:** January 2025

Databases Searched: PubMed, Scopus, Web of Science, MEDLINE, EMBASE, Dimensions

Time Frame: Inception to January 2025

**PubMed/MEDLINE Search Strategy**

((("shift work"[MeSH Terms] OR "shift work"[Title/Abstract] OR "shift worker*"[Title/Abstract] OR "night shift*"[Title/Abstract] OR "rotating shift*"[Title/Abstract] OR "day shift*"[Title/Abstract] OR "work schedule tolerance"[MeSH Terms] OR "night work"[Title/Abstract] OR "evening shift*"[Title/Abstract] OR "irregular shift*"[Title/Abstract] OR "alternating shift*"[Title/Abstract]))

AND (("exercise"[MeSH Terms] OR "exercise"[Title/Abstract] OR "physical activity"[Title/Abstract] OR "exercise therapy"[MeSH Terms] OR "physical fitness"[MeSH Terms] OR "motor activity"[MeSH Terms] OR "physical training"[Title/Abstract] OR "aerobic exercise"[Title/Abstract] OR "resistance training"[Title/Abstract] OR "strength training"[Title/Abstract] OR "endurance training"[Title/Abstract]) OR ("respiratory muscle training"[Title/Abstract] OR "inspiratory muscle training"[Title/Abstract]

OR "breathing exercises"[MeSH Terms] OR "breathing exercises"[Title/Abstract] OR "pulmonary rehabilitation"[MeSH Terms] OR "respiratory muscle strengthening"[Title/Abstract] OR "IMT"[Title/Abstract] OR "respiratory training"[Title/Abstract]))

AND (("sleep"[MeSH Terms] OR "sleep quality"[Title/Abstract] OR "sleep duration"[Title/Abstract] OR "sleep hygiene"[MeSH Terms] OR "sleep disorders"[MeSH Terms] OR "sleep disturbance*"[Title/Abstract] OR "sleep pattern*"[Title/Abstract] OR "sleep efficiency"[Title/Abstract] OR "sleep latency"[Title/Abstract] OR "circadian rhythm"[MeSH Terms] OR "circadian"[Title/Abstract]) OR ("cognition"[MeSH Terms] OR "cognitive function*"[Title/Abstract] OR "cognitive performance"[Title/Abstract] OR "neurocognitive"[Title/Abstract] OR "executive function"[MeSH Terms] OR "memory"[MeSH Terms] OR "attention"[MeSH Terms] OR "cognitive decline"[Title/Abstract] OR "cognitive impairment"[Title/Abstract])))

Filters: Humans; English language; Randomized Controlled Trial

**Scopus Search Strategy**

TITLE-ABS-KEY(("shift work" OR "shift worker*" OR "night shift*" OR "rotating shift*" OR "day shift*" OR "night work" OR "evening shift*" OR "irregular shift*" OR "alternating shift*") AND ("exercise" OR "physical activity" OR "exercise therapy" OR "physical fitness" OR "motor activity" OR "physical training" OR "aerobic exercise" OR "resistance training" OR "strength training" OR "endurance training" OR "respiratory muscle training" OR "inspiratory muscle training" OR "breathing exercise*" OR "pulmonary rehabilitation" OR "respiratory muscle strengthening" OR "IMT" OR "respiratory training") AND ("sleep quality" OR "sleep duration" OR "sleep hygiene" OR "sleep disorder*" OR "sleep disturbance*" OR "sleep pattern*" OR "sleep efficiency" OR "sleep latency" OR "circadian rhythm*" OR "circadian" OR "cognitive function*" OR "cognitive performance" OR "neurocognitive" OR "executive function*" OR "memory" OR "attention" OR "cognitive decline" OR "cognitive impairment"))

AND (LIMIT-TO (DOCTYPE, "ar") OR LIMIT-TO (DOCTYPE, "re"))

AND (LIMIT-TO (LANGUAGE, "English"))

**Web of Science Search Strategy**

TS= (("shift work" OR "shift worker*" OR "night shift*" OR "rotating shift*" OR "day shift*" OR "night work" OR "evening shift*" OR "irregular shift*" OR "alternating shift*") AND ("exercise" OR "physical activity" OR "exercise therapy" OR "physical fitness" OR "motor activity" OR "physical training" OR "aerobic exercise" OR "resistance training" OR "strength training" OR "endurance training" OR "respiratory muscle training" OR "inspiratory muscle training" OR "breathing exercise*" OR "pulmonary rehabilitation" OR "respiratory muscle strengthening" OR "IMT" OR "respiratory training") AND ("sleep quality" OR "sleep duration" OR "sleep hygiene" OR "sleep disorder*"

OR "sleep disturbance*" OR "sleep pattern*" OR "sleep efficiency" OR "sleep latency" OR "circadian rhythm*" OR "circadian"

OR "cognitive function*" OR "cognitive performance" OR "neurocognitive" OR "executive function*" OR "memory" OR "attention" OR "cognitive decline" OR "cognitive impairment")) Refined by: DOCUMENT TYPES: (Article OR Review) AND LANGUAGES: (English)

**EMBASE Search Strategy**

('shift work'/exp OR 'shift work':ti,ab OR 'shift worker*':ti,ab OR 'night shift*':ti,ab OR 'rotating shift*':ti,ab OR 'day shift*':ti,ab OR 'night work':ti,ab OR 'evening shift*':ti,ab OR 'irregular shift*':ti,ab OR 'alternating shift*':ti,ab) AND ('exercise'/exp OR 'exercise':ti,ab OR 'physical activity'/exp OR 'physical activity':ti,ab OR 'kinesiotherapy'/exp OR 'exercise therapy':ti,ab OR 'fitness'/exp OR 'physical fitness':ti,ab OR 'motor activity'/exp OR 'motor activity':ti,ab OR 'aerobic exercise':ti,ab OR 'resistance training':ti,ab OR 'strength training':ti,ab OR 'endurance training':ti,ab OR 'breathing exercise'/exp OR 'respiratory muscle training':ti,ab OR 'inspiratory muscle training':ti,ab OR 'breathing exercise*':ti,ab OR 'lung rehabilitation'/exp OR 'pulmonary rehabilitation':ti,ab OR 'respiratory muscle strengthening':ti,ab OR 'IMT':ti,ab OR 'respiratory training':ti,ab) AND ('sleep'/exp OR 'sleep quality'/exp OR 'sleep quality':ti,ab OR 'sleep duration':ti,ab OR 'sleep hygiene'/exp OR 'sleep disorder'/exp OR 'sleep disturbance*':ti,ab OR 'sleep pattern*':ti,ab OR 'sleep efficiency':ti,ab OR 'sleep latency':ti,ab OR 'circadian rhythm'/exp OR 'circadian':ti,ab OR 'cognition'/exp OR 'cognitive function*':ti,ab OR 'cognitive performance':ti,ab OR 'neurocognitive':ti,ab OR 'executive function'/exp OR 'memory'/exp OR 'attention'/exp OR 'cognitive decline':ti,ab OR 'cognitive defect'/exp OR 'cognitive impairment':ti,ab)AND [humans]/lim AND [english]/lim AND ([article]/lim OR [review]/lim)

**Dimensions Search Strategy**

("shift work" OR "shift worker" OR "shift workers" OR "night shift" OR "night shifts" OR "rotating shift" OR "rotating shifts" OR "day shift" OR "day shifts" OR "night work" OR "evening shift" OR "evening shifts" OR "irregular shift" OR "irregular shifts" OR "alternating shift" OR "alternating shifts")AND("exercise" OR "physical activity" OR "exercise therapy" OR "physical fitness" OR "motor activity" OR "physical training" OR "aerobic exercise" OR "resistance training" OR "strength training" OR "endurance training" OR "respiratory muscle training"

OR "inspiratory muscle training" OR "breathing exercises" OR "pulmonary rehabilitation" OR "respiratory muscle strengthening" OR "IMT" OR "respiratory training") AND ("sleep quality" OR "sleep duration" OR "sleep hygiene" OR "sleep disorder" OR "sleep disorders" OR "sleep disturbance" OR "sleep disturbances" OR "sleep pattern" OR "sleep patterns" OR "sleep efficiency" OR "sleep latency" OR "circadian rhythm" OR "circadian rhythms" OR "circadian" OR "cognitive function" OR "cognitive functions" OR "cognitive performance" OR "neurocognitive" OR "executive function" OR "executive functions" OR "memory" OR "attention" OR "cognitive decline" OR "cognitive impairment")

Publication Type: Article OR Review

Language: English
